# Bagasse as a carbon structure with high sulfur content for lithium–sulfur batteries†

**DOI:** 10.1039/d0ra01485k

**Published:** 2020-09-01

**Authors:** Jingjing Ma, Li Yang, Xiaoxun Yang, Yuanchao Li, Erqing Zhao, Shumin Fan, Guangri Xu, Tianjun Lou, Hongying Niu

**Affiliations:** College of Chemistry and Chemical Engineering, Henan Institute of Science and Technology Xinxiang 453003 P. R. China hongying_niu1974@163.com

## Abstract

A bagasse-based 3D carbon matrix (BC) with high specific surface area and high conductivity was obtained by carbonization and pore-forming processes with bagasse as the carbon precursor and K_2_FeO_4_ as the pore-former. The microporous structure and nitrogenous functional groups were determined in the prepared carbon matrix, which could allow high sulfur loading and improve the polysulfide absorption capacity during cycling. After sulfur infusion, the S/BC composite with 68.8% sulfur content was obtained. The lithium–sulfur (Li–S) battery with the S/BC cathode shows high specific capacity and good cycling performance. It delivers a specific capacity of 1360 mA h g^−1^ at 0.2C and remains at 790 mA h g^−1^ after 200 cycles. At 1C, the Li–S with this composite cathode exhibits 601 mA h g^−1^ after 150 cycles. This work offers a new kind of green material and a new method for Li–S batteries.

## Abstract 2

Bagasse-based carbon matrix with microporous structure and nitrogenous functional groups could have high sulfur loading and excellent polysulfide absorption capacity.

## Graphical abstract

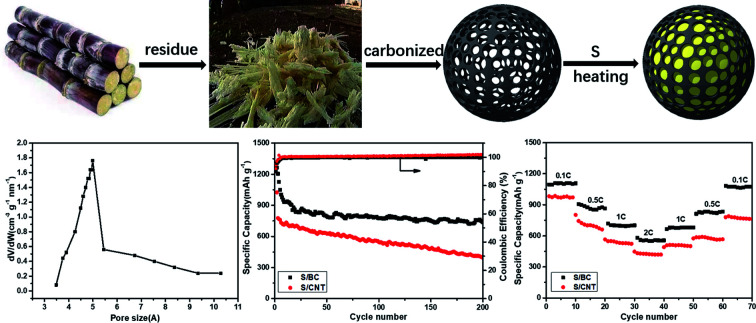

## Introduction

With the development of times and progress of science, wearable electronic devices, electric vehicles and new energy appliances demand more and more energy storage systems with high energy density and long service life.^1,2^ The lithium–sulfur battery has been considered as one of the most promising next generation energy storage systems due to its high specific capacity (1672 mA h g^−1^), high theoretical energy density (2600 W h kg^−1^) and low cost of sulfur.^3,4^ However, the application of Li–S batteries is prevented by a number of problems including low conductivity of sulfur element (5 × 10^−30^ S cm^−1^ at 25 °C) and the dissolution of lithium polysulfides.^5,6^ Many materials have been studied as the host for the sulfur loading, such as graphene,^7–9^ porous carbon,^10,11^ carbon sphere,^12,13^ carbon nanotubes,^14,15^ carbon nanofiber^16,17^ and other carbon composites.^18,19^ Although these studies have made some progresses, there is still a long way to go in the application of Li–S batteries.^20–22^

Many biomaterials are widely used as precursors to prepare porous carbon materials, such as rice,^23^ peanut shell,^24^ cotton^25,26^ and loofah sponge.^27^ Bagasse is the product leftover after sugar has been extracted from sugar cane, which is used to prepare carbon porous material. According to the test, this kind of carbon material has a high specific surface area and high electrical conductivity. The structure of this porous carbon material and functional groups which contain N and O elements could effectively reduce the shuttle action of polysulfide.^28^ The production of bagasse is enormous. It is suitable used to prepare the 3D porous carbon matrix (BC) for the Li–S battery. In addition, the bagasse is cheap and environmentally friendly.

## Materials and methods

### Preparation of porous bagasse carbon

The bagasse was got from a sugar refinery in Xinxiang, China. The bagasse was first shredded and washed with purified water to remove impurities. Then the bagasse was dipped into 1 M H_2_SO_4_ solution for 24 h and dried at 80 °C to remove the water. After that, the sample was heated at 400 °C for 2 h at the heating rate of 5 °C min^−1^ under nitrogen atmosphere to get bagasse carbon. K_2_FeO_4_ powder was dissolved in pure water and obtained a solution of 0.04 M concentration. Then the prepared bagasse carbon (0.5 g) was added into the solution under stirring. After 8 h, this sample was dried at 80 °C to remove the water. The dried material was heated at 800 °C for 2 h with heating rate of 5 °C min^−1^ in a tube furnace under nitrogen atmosphere. The cooled material was washed several times with dilute hydrochloric acid and purified water and dried to obtain bagasse-based porous carbon matrix (BC).

### Preparation of S/BC composite

The porous BC and sulfur were mixed evenly with a mass ratio of 1 : 5 using ball milling method. The mixture was heated at 155 °C for 20 h with the heating rate of 0.5 °C min^−1^, and next in the heating rate of 5 °C min^−1^ up to 300 °C and keep for 2 h under nitrogen atmosphere. After cooling, the S/BC was obtained. For comparison, carbon nanotubes (CNT) and sulfur elements were mixed at the same mass ratio. After a series of the same steps described above, the S/CNT composite material was obtained.

### Material characterization

The scanning electron microscopy (SEM) was recorded on a field emission SEM (Nova NanoSEM 450, FEI Company, USA). Fourier transform infrared (FTIR) spectra was measured by a Paragon 1000 spectrophotometer (PerkinElmer, Inc US). Raman spectrometry was performed using a Raman spectrometer (DXR, Thermo Fisher Scientific Inc., USA) with a laser wavelength of 532 nm at room temperature. The structure of the composite was tested by powder X-ray diffraction (D8 Advance, Bruker Corp., Germany, XRD) using Cu-Kα radiation at 40 kV. Electrical conductivity was done by the method of four-point probe (DP-SB100A/20, Beijing Ya'ou De Peng Technology Co. Ltd., China). Specific surface area and aperture distribution of the composites was performed by specific surface analyzer (ASAP 2010M + C, Micromeritics, USA).

### Electrochemical measurements

S/BC or S/CNT composite, Super-P and CMC were mixed with the mass ratio of 8 : 1 : 1 to homogeneous slurry and coated onto the aluminum foil and dried in a vacuum oven at 60 °C to fabricate working electrodes. The sulfur loading of the cathode was 1 mg cm^−2^. The coin cells were assembled in an argon-filled glove box (Mbraun, H_2_O, O_2_ < 0.1 ppm) with 1 M Li bis(trifluoromethane-sulfonamide)imide (LiTFSI) and 2 wt% LiNO_3_ in 1,3-dioxolane (DOL)/1,2-dimethoxyethane solvent (DME) (1 : 1, by volume) as electrolyte, Celgard 2400 as separator and Li foil (Alfa Aesar) as anode. The charge–discharge was tested by Land CT2001A multi-channel battery testing system within the voltage range from 1.7 V to 2.8 V (*vs.* Li/Li^+^) at room temperature. The rate performance was tested by the same testing system. Cyclic voltammetry (CV) was tested at the scan rate of 0.2 mV s^−1^ and the electrochemical impedance spectroscopy (EIS) was recorded within the frequency from 100 kHz to 0.1 Hz by CHI 660 Electrochemical Work Station (Shanghai Chenhua Group, China).

## Results and discussion

**Fig. 1:**
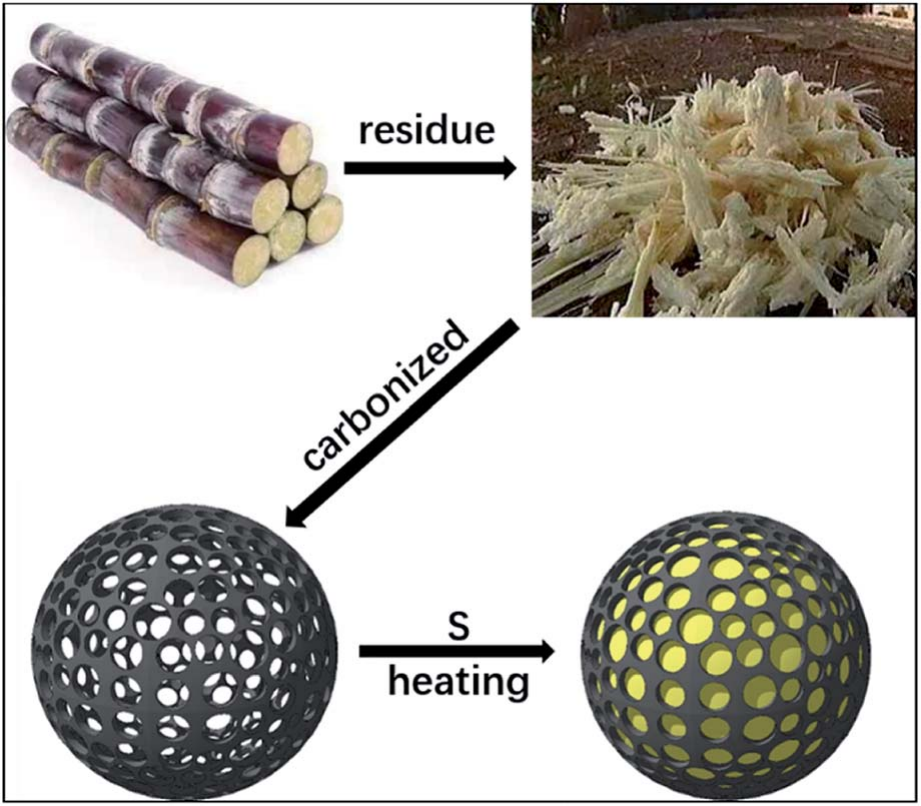
The preparation process of S/BC composite.

**Fig. 2:**
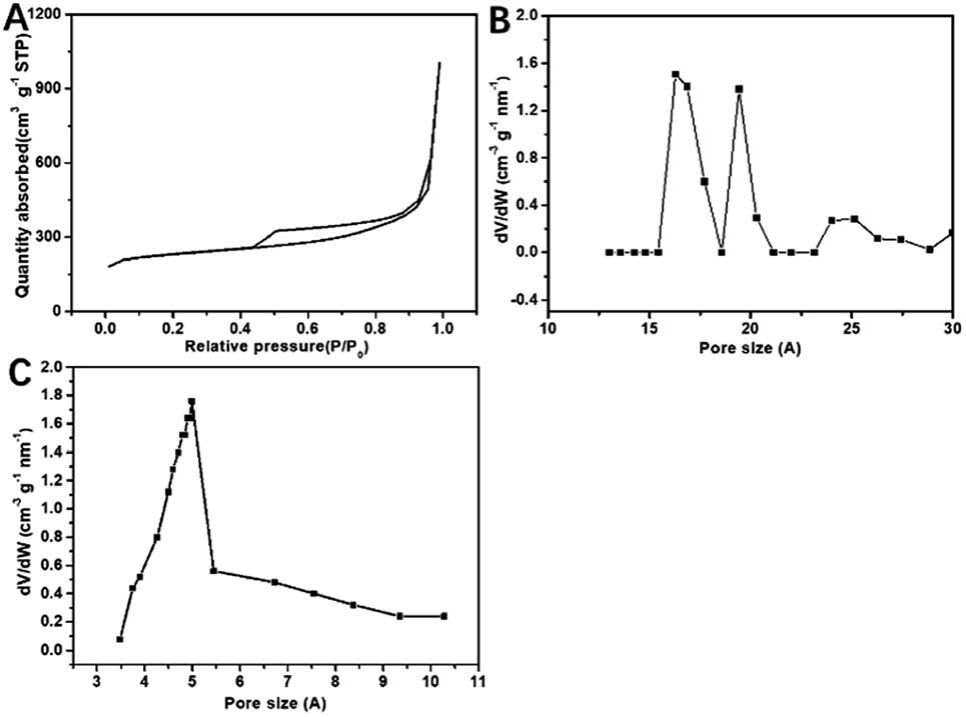
N_2_ adsorption/desorption isotherms of S/BC (A); pore size distribution plots calculated by H–K (B) and DFT of S/BC (C).

Fig. 1 shows the preparation of S/BC composite materials. In general, the porous carbon was prepared from bagasse and then heated with sulfur to get the cathode material. As the precursor, the bagasse was treated to make pores. After carbonized, the bagasse-based porous carbon matrix with high specific surface area was obtained. Through the test result of the nitrogen adsorption–desorption isotherms, the porous carbon matrix displays a isotherms of BET surface area of 793.4 m^2^ g^−1^ (Fig. 2A), which is much higher than that of the CNT (362.9 m^2^ g^−1^, Fig. S1A†). The pore size of this porous carbon matrix is 1.7–2 nm calculated by DFT method (Fig. 2B) and 0.5 nm calculated by Horvath–Kawazoe (H–K) method (Fig. 2C). In addition, the CNT has a pore size of 1–2 nm (calculated by DFT method, Fig. S1B†) and 0.9 nm (calculated by H–K method, Fig. S1C†).

**Fig. 3:**
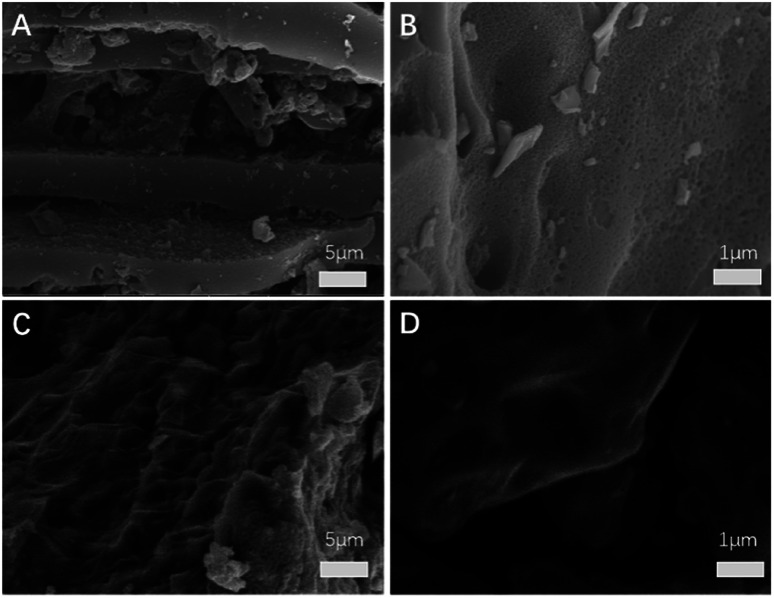
SEM images of BC (A and B) and S/BC (C and D).

The SEM images of BC and S/BC samples are shown in Fig. 3. The BC sample contains many pores which could provide space for sulfur loading and Li^+^ diffusion/transport (Fig. 3A and B). After mixed with the sulfur and heating, the pores were filled with the sulfur (Fig. 3C and D). As a result, the S/BC composite was obtained. In addition, energy-dispersive X-ray spectroscopy (EDS) analysis shows homogeneous distribution of S and C elements throughout the S/BC composite (Fig. S2†), suggesting the sulfur loaded within the BC matrix rather than aggregated on the surface.

**Fig. 4:**
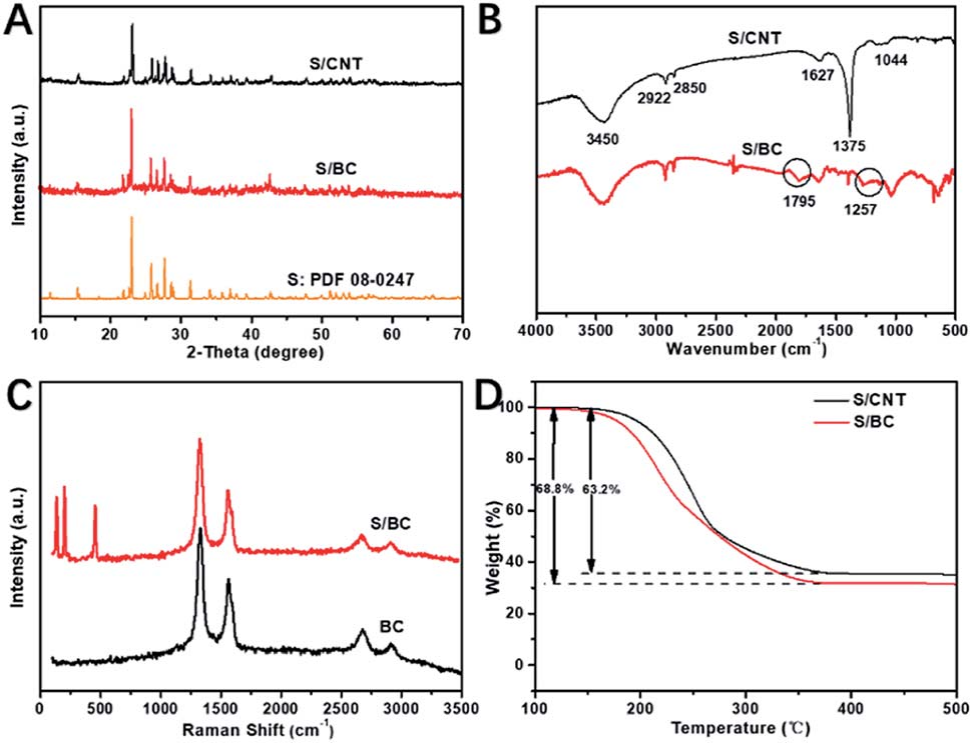
XRD patterns of S/CNT, S/BC and S (A); FTIR spectrum of S/BC and S/CNT (B); Raman spectra of BC and S/BC (C); TGA of S/BC and S/CNT (D).

X-ray diffraction (XRD) patterns of S/CNT, S/BC and S are shown in Fig. 4A. In Fig. 4A, the peaks of sulfur at 23°, 25°and 28° still remained in the XRD peaks of S/BC and S/CNT, suggesting that the sulfur is present in long chains. In Fig. 4B, FT-IR spectrum peaks at 1795 cm^−1^ and 1257 cm^−1^ in the S/BC represent the vibration strengths of C=N and C–N, respectively, which could not been found in the FTIR spectrum of S/CNT. It means the S/BC has the N element. As a result, these groups would have great effects on absorbing polysulfides. Fig. 4C shows the Raman spectra of BC and S/BC. Two peaks at 2700 cm^−1^ and 2900 cm^−1^ are due to the presence of graphitized carbon, while the peaks at 500 cm^−1^, 250 cm^−1^ and 200 cm^−1^ stand for sulfur. To make a comparison, the Raman spectra of CNT and S/CNT (Fig. S3†) composite were determined. The characteristic peaks mentioned above also appear in it. The graphitic carbon could improve the electron transport. Through four-point technique test, the electronic conductivity of S/BC is 0.83 S cm^−1^, and it is higher than some other S/porous carbon composites including S/CNT prepared in this paper (0.14 S cm^−1^). The theoretical maximum sulfur capacity is 74.4 wt%, which is based on the density of liquid sulfur (1.82 g cm^−3^) and the volume of pore (1.6 cm^3^ g^−1^). The content of sulfur in the S/BC and S/CNT composite was 68.8% and 63.2%, respectively by thermogravimetric analysis (TGA) test (Fig. 4D).

**Fig. 5:**
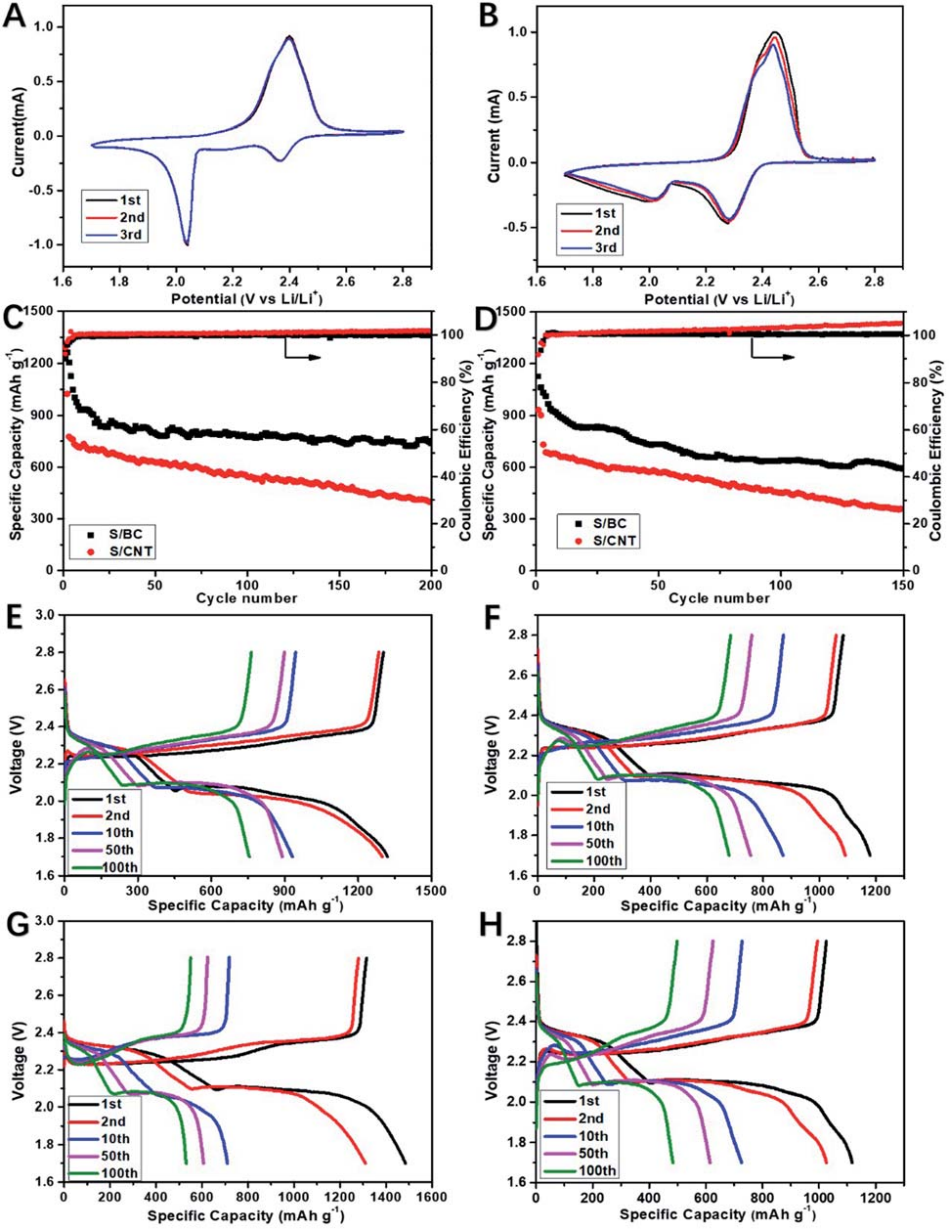
Cyclic voltammograms of the S/BC cell (A) and S/CNT cell (B); cycling performance of the S/BC cell and S/CNT cell at 0.2C (C) and 1C (D); discharge/charge profiles of the S/BC cell and S/CNT at 0.2C (E and F) and at 1C (G and H).

**Fig. 6:**
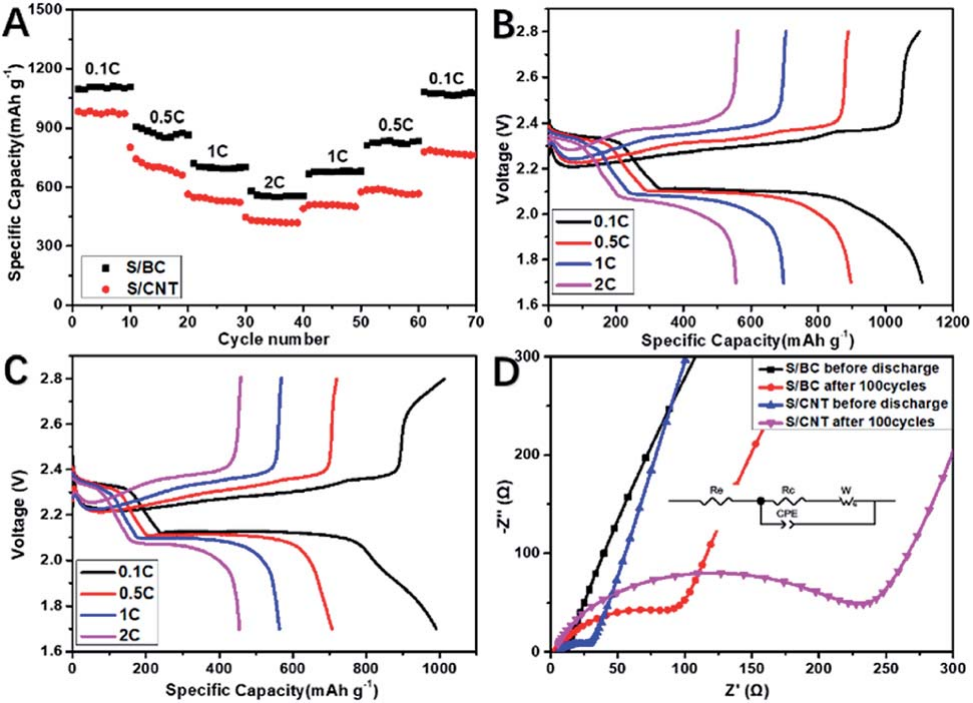
Rate performance of the S/BC cell and S/CNT cell (A); discharge/charge profiles of the S/BC (B) and S/CNT (C) at different current densities; EIS plots of the S/BC cell and S/CNT cell after different cycles at 0.2C (D).

Cyclic voltammograms (CVs) was used to study the kinetic process of S/BC and S/carbon nanotubes (S/CNT) cathode composites during charge and discharge. Fig. 5A and B show that the S/BC battery has better peak consistency than the S/CNT battery, indicating the S/BC battery has good cycle stability, which is due to the structure and functional groups of cathode composites in the S/BC battery. Both of these two batteries have two reduction peaks corresponding to the stepwise reduction from S_8_ to long chain ordered polysulfides (Li_2_S_*y*_, 4 ≤ *y* ≤ 8) and further to insoluble Li_2_S_2_/Li_2_S. The oxidation peak at 2.35–2.45 V represents the reversible conversion of lithium sulfide to metallic Li and S_8_. The electrochemical cycling performances of the S/BC battery and the S/CNT battery at 0.2C (Fig. 5C) and 1C (Fig. 5D) were evaluated. In the initial cycle, the S/BC exhibits a high discharge capacity of 1360 mA h g^−1^, and while after 200 cycles, it remains 790 mA h g^−1^ at 0.2C, which shows good cycle stability. On the contrary, the S/CNT battery demonstrates a low specific capacity (1048 mA h g^−1^, 1^st^ cycle) and a rapid capacity attenuation during the cycling (381 mA h g^−1^, 200^th^ cycle) when compared with the S/BC battery (Fig. 5E and F). At a higher rate, 1C, the S/BC battery also displays better electrochemical performance than the S/CNT cell (Fig. 5G and H). The coulombic efficiency of S/BC keeps a high stability near 100% during the cycling. For the S/CNT battery, it exceeds 100 percent and increases gradually because of the shuttle effect of lithium polysulfides. The results show that the bagasse-based porous carbon matrix could reduce polysulfide shuttle effectively due to its high electronic conductivity (8.2 S cm^−1^), porous structure and nitrogenous functional groups. Therefore the S/BC battery has better cycling performance than the S/CNT cell. Compared with the various carbon derived from natural materials for Li–S battery applications published recently (Table S1†), the cathode material S/BC synthesised in this work has advantages in reversible specific capacity and capacity retention. Rate performance profiles at different rate are shown in Fig. 6A. The discharge capacity is determined to be 1096 mA h g^−1^, 870 mA h g^−1^, 689 mA h g^−1^, and 555 mA h g^−1^ at 0.1, 0.5, 1 and 2C, respectively. Both S/BC and S/CNT cells indicate discharge capacity reduction when the discharge rate increases from 0.1C to 2C. The capacity recovery of S/BC is better than that of S/CNT when the discharge rate returned from 2C to 0.1C (Fig. 6B and C). The high rate capacity of S/BC is still the result of the porous structure, high electronic conductivity and groups in the cathode.

Electrochemical impedance spectroscopy (EIS) was measured to the change of electrode before and after circulation. The Nyquist plots of the S/BC cell and S/CNT cell at fully charged after different cycles are shown in Fig. 6D. From high frequency to middle, the plots display as a single semicircle, and in the low frequency region, it becomes a slope line, which represents interfacial charge transfer and lithium ion diffusion, respectively. The high frequency intercept represents the resistance of the battery, while the length between the high and low frequency intercepts represents the electrode polarization resistance. The Nyquist plots and equivalent circuits are consisted of the bulk resistance of the electrolyte (*R*_e_), charge transfer resistance (*R*_c_), constant phase element (CPE) and Warburg impedance (*W*). As shown in Table S2,† no matter at 1^st^ or 100^th^ cycle, the *R*_c_ for S/BC, the cell resistance and polarization resistance of S/BC batteries are both smaller than those of S/CNT batteries. This is due to the special structure of the bagasse-based porous carbon matrix.

## Conclusions

A 3D carbon matrix has been prepared using bagasse as carbon precursor and K_2_FeO_4_ as pore-former. The bagasse-based carbon (BC) possesses high specific surface area (793.4 m^2^ g^−1^), high conductivity (8.2 S cm^−1^), abundant micro pores (pore width < 2 nm, pore volume 1.6 cm^3^ g^−1^) and nitrogenous functional groups, which make it a good carbon host for sulfur loading. Among them, the porous structure and nitrogenous functional groups in BC could allow high sulfur loading and effectively inhibit lithium polysulfide shuttle. As a result, the S/BC cathode composite with 68.8% sulfur content was obtained and exhibits superior long-term cycling stability and rate performance in S/BC|Li full cell. It delivers high specific capacity of 1360 mA h g^−1^ at 0.2C in the initial cycle and remains 790 mA h g^−1^ after 200 cycles with a stable coulombic efficiency near 100% during cycling. At 1C, the Li–S with this composite cathode exhibits 601 mA h g^−1^ after 150 cycles. This work provides a good idea for the preparation of low cost and green Li–S battery.

## Conflicts of interest

There are no conflicts to declare.

## Supplementary Material

RA-010-D0RA01485K-s001
