# Supplementary material for: Bagasse as a carbon structure with high sulfur content for lithium–sulfur batteries
Source: RSC Adv. 2020 Sep 1;10(54):32345–9. doi: 10.1039/d0ra01485k (PMC9056595; doi:10.1039/d0ra01485k)
Supplement: RA-010-D0RA01485K-s001 [file RA-010-D0RA01485K-s001.pdf]

Electronic Supplementary Material (ESI) for RSC Advances.
This journal is © The Royal Society of Chemistry 2020

Begasse as a carbon structure with high sulfur content for lithium-sulfur battery

Jingjing Ma[a], Li Yang[a], Xiaoxun Yang[a], Yuanchao Li[a], Erqing Zhao[a], Shumin Fan[a], Guangri Xu[a], Tianjun Lou[a], and Hongying Niu*[a]

College of Chemistry and Chemical Engineering, Henan Institute of Science and Technology, Xinxiang, 453003, P. R. China.

Corresponding author: Hongying Niu

E-mail address: hongying_niu1974@163.com

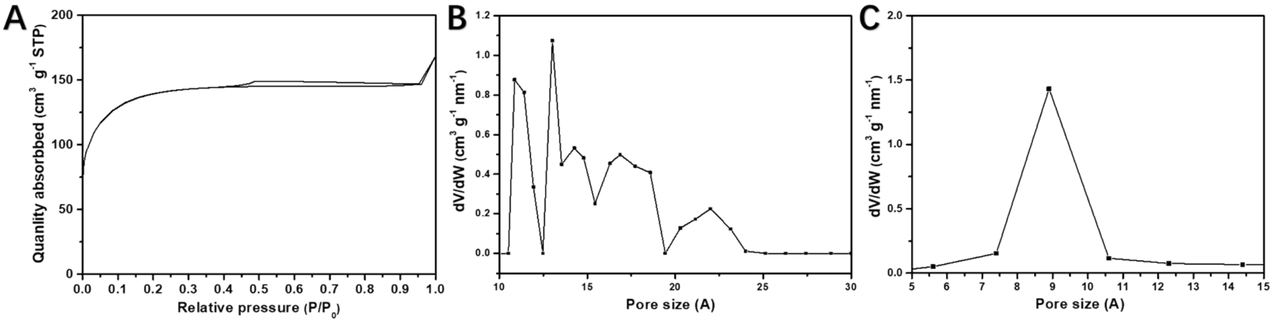

Fig.S1 N_2 adsorption/desorption isotherms (A) of S/CNT; Pore size distribution plots calculated by H-K (B) and DFT (C) of S/CNT

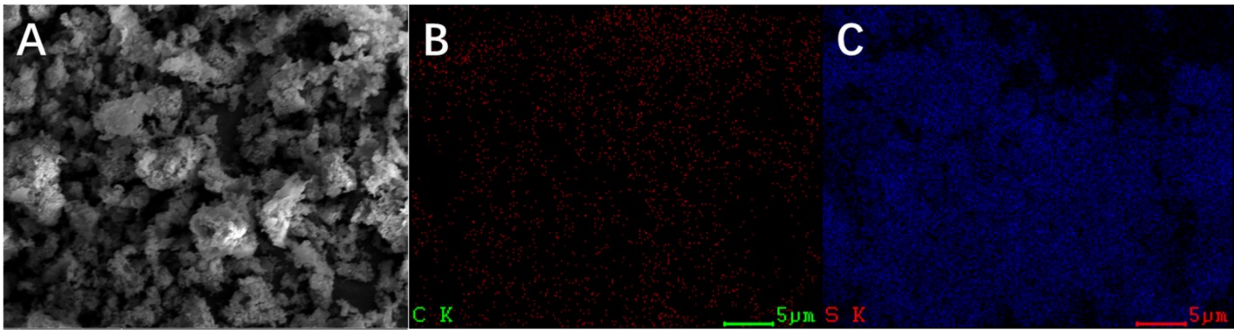

Fig.S2 SEM images of S/BC (A) and the corresponding C (B) and S (C) elemental mappings

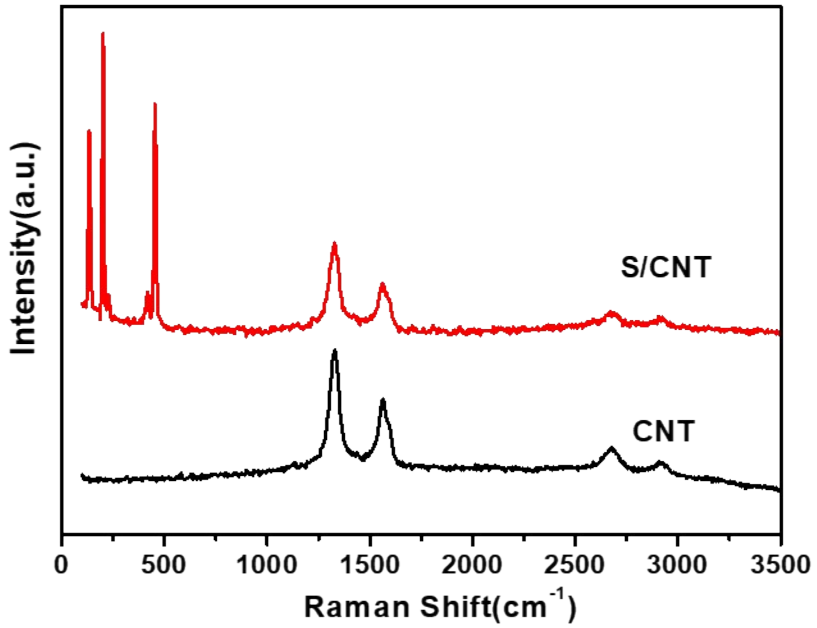

Fig.S3 Raman spectra of CNT and S/CNT

Table S1. Summary of some publications of natural materials used for the Li-S battery

Sample name	Sulfur Content (wt%)	First reversible specific capacity (mAh/g)	Capacity retention (mAh/g)	Cycle number	Ref
S/NMPC	63	1327	757	100(0.1C)	[1]
S/NMPC	63	1327	624	100(1C)	[1]
S/NBC-15	56	1169	898	200(0.2C)	[2]
S/NBC-15	56	1098	682	500(0.5C)	[2]
Pomelo peel carbon	60	1258	750	100(0.2C)	[3]
Starch derived carbon	81.29	922	683	100(0.5C)	[4]
Spent coffee carbon	47.6	1150	613	100(0.2C)	[5]
N/BC	68.8	1360	790	200(0.2C)	This work
N/BC	68.8		601	150(1C)	This work

Table S2. The Nyquist plots of S/BC and S/CNT

Conditions and parameters		S/BC	S/CNT
At the 1st	**Re(Ω)**	2.9	2.9
	**Rc(Ω)**	11.3	28.7
At the 10th	**Re(Ω)**	3.4	3.5
	**Rc(Ω)**	94.2	277.5

References

1. Y. Zhao, X. Zhang, Y. He, N. Liu, T. Tan and C. Liang, *Materials*, 2017, **10**, 1158.
2. D. B. Babu and K. Ramesha, *Carbon*, 2019, **144**, 582-590.
3. J. Zhang, J. Xiang, Z. Dong, Y. Liu, Y. Wu, C. Xu and G. Du, *Electrochimica Acta*, 2014, **116**, 146-151.
4. J. Li, F. Qin, L. Zhang, K. Zhang, Q. Li, Y. Lai, Z. Zhang and J. Fang, *Journal of Materials Chemistry A*, 2014, **2**, 13916-13922.
5. Y. Zhao, L. Wang, L. Huang, M. Maximov, M. Jin, Y. Zhang, X. Wang and G. Zhou, *Nanomaterials*, 2017, **7**, 402.
